# Possible selection bias in register-based obesity studies

**DOI:** 10.1007/s10654-025-01237-6

**Published:** 2025-05-12

**Authors:** Lena MS Carlsson, Markku Peltonen, Peter Jacobson, Johanna C Andersson-Assarsson, Per-Arne Svensson, Magdalena Taube, Cecilia Karlsson, Sofie Ahlin, Felipe M Kristensson, Rosie Perkins, Ida Arnetorp, Alexander Carlsson, Lucas Admeus, Elin Langegård, Björn Carlsson, Kajsa Sjöholm

**Affiliations:** 1 Institute of Medicine, Sahlgrenska Academy, University of Gothenburg, Gothenburg, Sweden; 2 Finnish Institute for Health and Welfare, Helsinki, Finland; 3 Institute of Health and Care Sciences, Sahlgrenska Academy, University of Gothenburg, Gothenburg, Sweden; 4 Late-Stage Development, Cardiovascular, Renal and Metabolism (CVRM), BioPharmaceuticals R&D, AstraZeneca, Gothenburg, Sweden; 5 Department of Clinical Physiology, Region Västra Götaland, NU hospital group, Trollhättan, Sweden; 6 Dept of Surgery, Region Västra Götaland, Sahlgrenska University Hospital/Östra, Gothenburg, Sweden; 7 BioPharmaceuticals R&D, AstraZeneca, Research and Early Development, Cardiovascular, Renal and Metabolism (CVRM), Gothenburg, Sweden

**Keywords:** Obesity diagnosis, Register-based studies, Life expectancy

## Abstract

Some studies of obesity treatments use control groups identified from real-world registers, which may differ from people with obesity in the general population. We evaluated whether such control groups affect the results. The SOS study examines long-term mortality post-bariatric surgery. Among volunteers with obesity, 2,007 individuals underwent surgery, while a control group of 2,040 individuals was matched using 18 variables. Age was 37–60 years and BMI was ≥ 34/≥38 kg/m^2^ for men and women, respectively. We subdivided the control group into those with an obesity diagnosis (*n* = 177) and those without an obesity diagnosis (*n* = 1,863) in the Swedish National Patient Register prior to study inclusion. Mortality was determined over a median follow-up period of 26 years. The controls with a prior obesity diagnosis had a higher mortality rate than the controls without a prior obesity diagnosis, with 19.7 (95% CI, 15.5–25.1) and 14.4 (95% CI, 13.3–15.7) deaths per 1000 person-years, respectively. This corresponds to a hazard ratio of 1.45 (95% CI, 1.12–1.89; *p* = 0.005) and a 3.4-year shorter life expectancy. These results were confirmed in another cohort (*n* = 2,759, HR = 1.82 [95% CI, 1.47–2.25; p<0.001] and a 6.1-year shorter life expectancy). Controls with obesity identified from real-world datasets may be in poorer health than those who voluntarily participate in clinical studies. Consequently, selection bias could lead to an overestimation of the survival benefits of obesity treatments in research using controls identified by prior obesity diagnosis.

**Supplementary Information:**

The online version contains supplementary material available at 10.1007/s10654-025-01237-6.

## Introduction

Obesity is associated with increased incidence of serious conditions such as cardiovascular disease and cancer and shortens life span by 5–20 years.[1, 2, 3] Bariatric surgery is an established treatment of obesity and results in large and maintained weight loss,[4] but its long-term effects on mortality have not been examined in large randomized controlled trials (RCTs). Current knowledge is therefore to a large extent based on retrospective studies where patients treated by bariatric surgery have been compared to controls identified by an obesity diagnosis in real-world datasets such as electronic health records and health registers.[5] The only prospective study is the matched Swedish Obese Subjects (SOS) study, which was designed to examine overall mortality after bariatric surgery compared to usual obesity care. Results from the SOS study have shown an association between bariatric surgery and a reduced relative risk of mortality,[6] with a median life expectancy approximately 3 years longer compared to matched controls receiving usual obesity care.[7] However, a recent meta-analysis estimated that bariatric surgery increases life expectancy by 6 years.[5].

It is unclear why the estimated median survival benefit after bariatric surgery is much greater in the meta-analysis than in the SOS study but one explanation may be study design. In the SOS study, participants with obesity were recruited voluntarily through mass media campaigns and at primary healthcare centers. The meta-analysis included the prospective SOS study but was primarily dominated by 16 retrospective studies that used real-world datasets to identify individuals with obesity for both the control and surgery groups. While individuals who undergo bariatric surgery must meet specific criteria for BMI and comorbidities and be healthy enough for surgery, much less is known about the health status of those included in control groups. The current study aimed to test the hypothesis that the methods used to define control groups may influence the estimated outcomes in studies of bariatric surgery.

## Methods

### Study design

Between 1987 and 2001, the SOS study enrolled 4047 individuals with obesity.[7, 8] In brief, 6,905 individuals, recruited through mass media campaigns and at primary healthcare centers, participated in a matching examination, forming the SOS matching cohort. From this cohort, 4,047 participants were selected for the SOS intervention study (see flowchart in Supplementary Figure S1). In the intervention study, individuals who chose surgical treatment (*n* = 2,007) formed the surgery group and a non-randomized contemporaneously matched control group (*n* = 2,040) was created using 18 matching variables according to the method of sequential treatment assignment.[9] The matching variables were sex, age, smoking, diabetes, weight, height, waist circumference, hip circumference, systolic blood pressure, triglycerides, total cholesterol, pre/postmenopausal, current health, monotony avoidance, psychasthenia, quantity of social support, quality of social support and stressful life events. The surgery and control groups had identical inclusion and exclusion criteria and all participants in both groups were eligible for surgery. The inclusion criteria were age 37 to 60 years and BMI of 34 kg/m^2^ or more for men and 38 kg/m^2^ or more for women. The exclusion criteria were earlier surgery for gastric or duodenal ulcer, earlier bariatric surgery, gastric ulcer in the past 6 months, ongoing malignancy, active malignancy in the past 5 years, myocardial infarction in the past 6 months, bulimic eating pattern, drug or alcohol abuse, psychiatric or cooperative problems contraindicating bariatric surgery, other contraindicating conditions (such as chronic glucocorticoid or anti-inflammatory treatment). The study protocol was approved by seven regional ethics review boards and informed consent was obtained from all participants. The cut-off date for the current analysis was December 31, 2020. The study is registered at ClinicalTrials.gov, NCT01479452 (retrospectively registered November 22, 2011).

The intervention study was conducted at 480 primary healthcare centers and 25 surgical departments in Sweden. Baseline characteristics, including body weight, height, blood pressure, biochemical variables and electrocardiography data, were measured approximately 4 weeks before the start of the intervention. Blood samples were taken after an overnight fast. The study began on the day of surgery for individuals in the surgery group as well as for their matched individual in the control group. In both the surgery and control groups, physical examinations were performed and questionnaires were completed at baseline and after 0.5 and 1, 2, 3, 4, 6, 8, 10, 15 and 20 years. Urine and fasting blood samples obtained at baseline and after 2, 10, 15 and 20 years were analysed at the Central Laboratory, Sahlgrenska University Hospital, Gothenburg, Sweden, accredited according to International Organization for Standardization/International Electrochemical Commission 15189:2007 standards. Individuals in the surgery group underwent gastric bypass (*n* = 266), vertical banded gastroplasty (*n* = 1,365) and gastric banding (*n* = 376). The control group received the customary treatment of obesity at their primary healthcare centers.

Among the individuals in the SOS matching cohort who were not included in the intervention study, 2,759 participants who had not previously undergone bariatric surgery constituted the excess candidate pool (see flowchart in Supplementary Figure S1). Since individuals in the excess candidate pool did not participate in the baseline and follow-up examinations conducted in the intervention study, and were only monitored through health register data, their baseline characteristics were derived from information gathered during the matching examination. Furthermore, this cohort was not subject to the exclusion criteria applied to the surgery and control groups in the SOS intervention study, which were designed to select individuals healthy enough to undergo bariatric surgery.

### Register data

The SOS database was cross-linked to Swedish national health registers. Information on diagnoses was obtained from the National Patient Register using International Classification of Diseases (ICD) codes. This register has 99% coverage of inpatient care visits and around 80% coverage of specialist outpatient care visits for somatic diseases.[10] Information on deaths was obtained from the Swedish Population and Address Register (SPAR). Virtually 100% of births and deaths, 95% of immigrations and 91% of emigrations are reported to SPAR within 30 days.[11] Information on the official cause of death was obtained from the Swedish Cause of Death Register, an essentially complete register of all official (underlying) cause of deaths in Sweden since 1952.[12] The official cause of death reflects the underlying preventable cause (e.g., obesity) leading to death; relevant case sheets and autopsy reports were therefore assessed independently by two authors to determine the direct cause of death. When the official and direct cause of death differed, the direct cause of death was used. Study participants who emigrated, withdrew consent or were alive at the end of follow-up were censored on the corresponding date.

### Stratification of the control group based on prior obesity diagnosis

For the current study, participants of the control group who were diagnosed with obesity, as main or secondary diagnosis, before study inclusion in the SOS study were identified from the Swedish National Patient Register (ICD-8 277; ICD-9 278A and 278B; ICD-10 E66). The control group was then subdivided into two groups: controls with a prior obesity diagnosis (i.e., with an obesity diagnosis before inclusion) and controls without a prior obesity diagnosis. The same subgrouping was applied to the individuals of the excess candidate pool, which was used to verify the results of the SOS control group.

### Statistical analyses

Analyses were done per-protocol, censoring participants in the control group who underwent bariatric surgery and participants in the surgery group who underwent surgical intervention to restore normal anatomy at the time of this surgery. Baseline characteristics are presented as mean values with standard deviations or as percentages, and statistical significances were evaluated with an analysis of covariance for continuous variables and with Fisher’s exact test for dichotomous variables.

Kaplan–Meier estimates of survival functions were used to estimate cumulative mortality in controls with and without prior obesity diagnosis as well as in the surgery group, and the Gompertz proportional-hazards regression model was used to estimate differences in median survival times, as previously described.[7] Analyses of cause-specific mortality were conducted with the competing-risks regression models suggested by Fine and Gray[13] in which deaths for other reasons were treated as competing events.

Analyses were adjusted for age and sex (because this is common in studies using real-world data), and in additional analyses for BMI. All statistical tests were two-sided, and P values of less than 0.05 were considered to indicate statistical significance. Stata software, version 18.0 (StataCorp), was used for all analyses.

## Results

We identified 177 (8.7%) individuals in the SOS study control group who had an obesity diagnosis before study start (see flowchart in Supplementary Figure S1). Baseline characteristics of the controls with and without a prior obesity diagnosis are shown in Table 1. Several risk factors were less favourable in the controls with a prior obesity diagnosis, including higher concentrations of fasting blood glucose, serum insulin, HOMA-IR and prevalence of type 2 diabetes, and worse self-rated health status. In addition, baseline BMI was higher in the controls with a prior obesity diagnosis compared to the controls without a prior obesity diagnosis (41.8 ± 5.0 and 40.0 ± 4.7 kg/m^2^, respectively, *p* < 0.001). This difference persisted during follow-up (BMI 10 years after study inclusion: 43.0 and 41.0 kg/m^2^ in controls with and without a prior obesity diagnosis, respectively; difference 2.0 [95% CI, 0.8–3.1] kg/m^2^, *p* = 0.001).

**Table 1:** Baseline characteristics of the SOS study control group stratified by prior obesity diagnosis in the Swedish National patient register

	Controls with a prior obesity diagnosis (*n* = 177)	Controls without a prior obesity diagnosis (*n* = 1863)	*p*-value
Age - years	49.1 ± 5.9	48.7 ± 6.3	0.320
Male sex – no. (%)	42 (23.7)	551 (29.6)	0.119
Body mass index – kg/m^2^	41.8 ± 5.0	40.0 ± 4.7	< 0.001
Waist-to-hip ratio	0.993 ± 0.075	0.976 ± 0.073	0.005
Hypertension – no. (%)†	124 (70.1)	1177 (63.2)	0.072
Type 2 diabetes – no. (%)°	51 (28.8)	212 (11.4)	< 0.001
Blood glucose - mmol/L	5.9 ± 2.8	4.8 ± 1.7	< 0.001
Insulin – mU/L	19.9 ± 10.3	17.9 ± 11.5	0.024
HOMA-IR	6.1 ± 5.0	4.6 ± 4.4	< 0.001
HbA1c – mmol/mol	46.6 ± 16.5	40.0 ± 10.3	< 0.001
HDL-cholesterol - mmol/L	1.4 ± 0.4	1.3 ± 0.3	0.357
Total-cholesterol – mmol/L	5.7 ± 1.1	5.6 ± 1.1	0.482
LDL-cholesterol – mmol/L	3.3 ± 1.0	3.4 ± 0.9	0.519
Triglycerides – mmol/L	2.2 ± 1.7	2.0 ± 1.4	0.043
Daily smoking – no. (%)	47 (26.6)	375 (20.2)	0.053
Alcohol consumption – g/day	4.4 ± 8.5	5.4 ± 8.0	0.122
Self-rated health status*	3.6 ± 1.4	3.3 ± 1.3	0.007
Cancer before baseline – no. (%)	3 (1.7)	19 (1.0)	0.431
Cardiovascular disease before baseline – no. (%)	4 (2.3)	45 (2.4)	1.000

*Higher scores represent worse self-rated health status according to a graded scale (range 1–7)

°Based on fasting blood glucose level ≥ 6.1 mmol/L and/or use of anti-diabetes medication

† Defined as diastolic blood pressure > 90 mm Hg, systolic blood pressure > 140 mm Hg, or self-reported antihypertensive medication

**Fig. 1:**
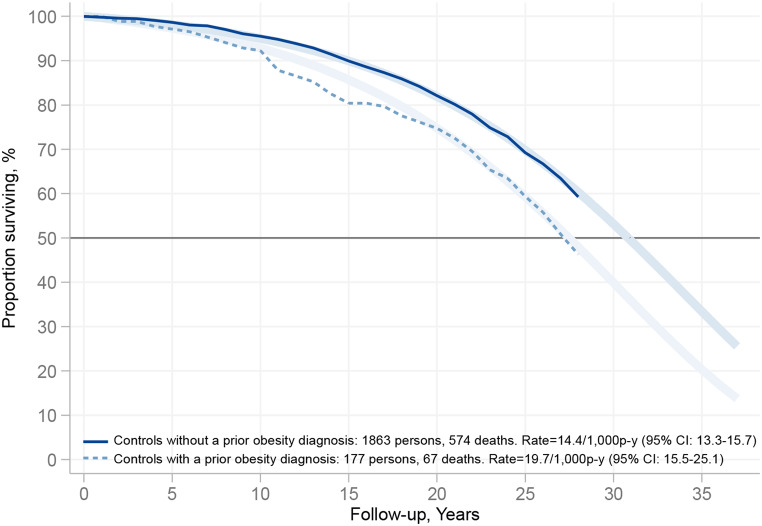
Survival in the SOS control group stratified by prior (before study inclusion) obesity diagnosis in the National Patient Register. Shown are Kaplan–Meier estimate of survival (opaque lines) and the estimate of survival from an unadjusted Gompertz regression model extrapolated up to 37 years (fainter lines)

During follow-up, there were 641 deaths in the control group. Of these, 67 occurred in the subgroup with a prior obesity diagnosis and 574 in the subgroup without a prior obesity diagnosis, corresponding to 19.7 (95% confidence interval [CI], 15.5–25.1) and 14.4 (95% CI, 13.3–15.7) deaths per 1,000 person-years, respectively (Table 2). Unadjusted mortality was higher in the controls with a prior obesity diagnosis (hazard ratio [HR], 1.45 [95% CI, 1.12–1.89] *p* = 0.005) and similar results were obtained after adjustment for age and sex, as well as after additional adjustment for BMI (Table 2). Figure 1 shows survival in the control groups with and without a prior obesity diagnosis. Unadjusted median survival time was 3.4 (95% CI, 1.0-5.7) years shorter in the controls with a prior obesity diagnosis compared to the controls without a prior obesity diagnosis. Similar results were obtained after adjustment for age and sex, as well as after additional adjustment for BMI (Table 2).

**Table 2:** Mortality rates, hazard ratios and differences in median life expectancy, from Gompertz proportional hazard regression model, in the SOS study control group stratified by prior obesity diagnosis

	Controls with a prior obesity diagnosis	Controls without a prior obesity diagnosis
n	177	1863
Person-time (years)	3,395	39,776
Events	67	574
Mortality rate	19.7	14.4
95% CI	15.5–25.1	13.3–15.7
HR (unadj)	1.45	Ref.
95% CI	1.12–1.89	
p-value	0.005	
HR (adj#)	1.49	Ref.
95% CI	1.15–1.92	
p-value	0.002	
HR (full adj*)	1.44	Ref.
95% CI	1.11–1.86	
p-value	0.006	
Difference in median survival time, years (unadj)	-3.4	Ref.
95% CI	-5.7 to -1.0	
Difference in median survival time, years (adj#)	-3.4	Ref.
95% CI	-5.6 to -1.2	
Difference in median survival time, years (full adj*)	-3.1	Ref.
95% CI	-5.3 to -0.9	

# Adjusted for age and sex

* Adjusted for age, sex and BMI

**Fig. 2:**
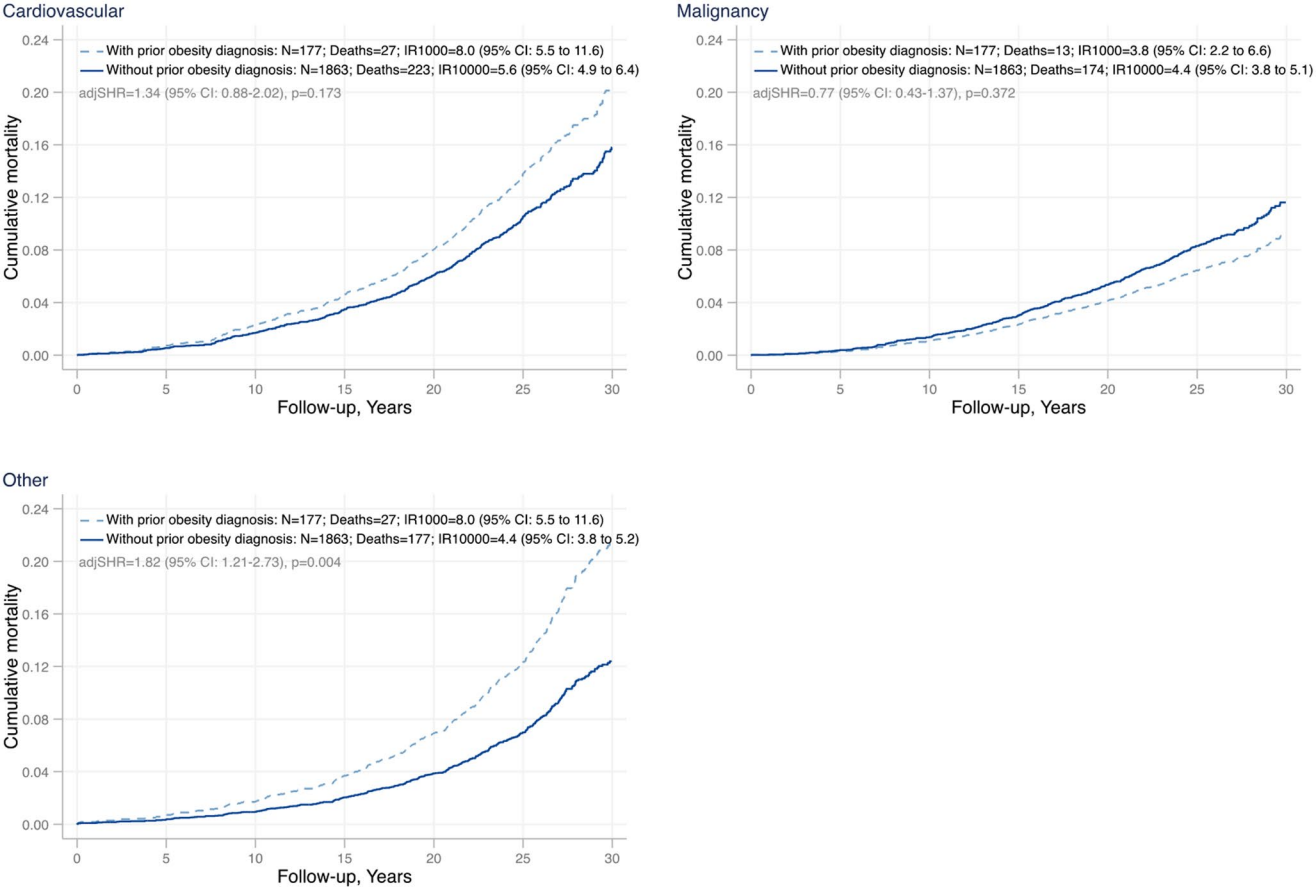
Cause-specific mortality in the SOS control group stratified by prior (before study inclusion) obesity diagnosis in the National Patient Register. Shown are deaths due to cardiovascular disease, malignancies and other causes. Controls with a prior obesity diagnosis are represented by dashed light blue lines. Controls without a prior obesity diagnosis are represented by solid dark blue lines. SHRa: sub-hazard ratio, adjusted for age, sex and BMI

The differences in overall mortality and life expectancy between individuals with and without a prior obesity diagnosis in the SOS control group were confirmed in the excess candidate pool. Baseline characteristics for this cohort are shown in Supplementary Table S1. Among the excess candidates, there were 1,040 deaths, and of these, 92 occurred in the subgroup with a prior obesity diagnosis and 948 in the subgroup without a prior obesity diagnosis, corresponding to 28.2 (95% CI, 23.0-34.6) and 17.2 (95% CI, 16.1–18.3) deaths per 1,000 person-years, respectively. Unadjusted mortality was higher in the excess candidates with a prior obesity diagnosis (HR, 1.82 [95% CI, 1.47–2.25] *p* < 0.001) and similar results were obtained after adjustment for age and sex (adjHR, 1.99 [95% CI, 1.59–2.49] *p* < 0.001), as well as after additional adjustment for BMI (adjHR, 1.57 [95% CI, 1.25–1.98] *p* < 0.001). Unadjusted median survival time was 6.1 (95% CI, 3.9–8.3) years shorter in the excess candidates with a prior obesity diagnosis compared to the those without a prior obesity diagnosis, 6.7 (95% CI, 4.6–8.9) shorter after adjustment for age and sex and 4.3 (95% CI, 2.2–6.5) years shorter after additional adjustment for BMI (Supplementary Table S2, Supplementary Figure S2). Cardiovascular disease was the most common specific cause of death in the SOS control group (Supplementary Table S3). While not significant, the incidence rate (IR) of cardiovascular mortality was higher in the controls with than in those without a prior obesity diagnosis (IR, 8.0 [95% CI, 5.5–11.6] versus 5.6 [95% CI, 4.9–6.4]; adjusted sub-hazard ratio [SHRa], 1.34 [95% CI, 0.88–2.02], *p* = 0.173) (Fig. 2). The incidence of deaths due to malignancies, the second most common cause of death, was similar in the controls with and without a prior obesity diagnosis (IR, 3.8 [95% CI, 2.2–6.6] and 4.4 [95% CI, 3.8–5.1], respectively; SHRa, 0.77 [95% CI, 0.43–1.37]; *p* = 0.372), while deaths due to other causes were more common in the controls with than in those without a prior obesity diagnosis (IR, 8.0 [95% CI, 5.5–11.6] versus 4.4 [95% CI, 3.8–5.2], respectively; SHRa, 1.82 [95% CI, 1.21–2.73], *p* = 0.004) (Fig. 2). Other causes of death included infections, neurological diseases, diseases of internal organs, thromboembolic diseases, multiple conditions and causes other than disease (Supplementary Table S3).

We next compared the SOS study surgery group with the SOS controls with and without prior obesity diagnosis to assess possible implications of using control groups created by different methods to study outcomes after bariatric surgery. Baseline characteristics of this group are shown in Supplementary Table S4. After bariatric surgery, BMI was markedly reduced, with a nadir at 1 year followed by partial weight regain (Supplementary Figure S3). During follow-up, there were 559 deaths in the surgery group. After adjustment for age, sex and BMI, mortality in the controls with a prior obesity diagnosis was higher than in the surgery group (HR, 1.65 [95% CI, 1.27–2.15]; *p* = 0.001) and median survival time was 4.3 (95% CI, 2.1–6.5) years shorter (Supplementary Table S5, Supplementary Figure S4A). In the SOS controls without a prior obesity diagnosis, mortality was also higher than in the surgery group (HR, 1.15 [95% CI, 1.02–1.30]; *p* = 0.024) but the difference was smaller than for the controls with prior obesity diagnosis (test of equal HRs *p* = 0.006) and the median survival time was 1.2 (95% CI, 0.2–2.3) years shorter than in the surgery group (Supplementary Table S5, Supplementary Figure S4B).

## Discussion

To assess the potential impact of a prior obesity diagnosis on clinical outcomes in obesity studies, we divided participants in the SOS study control group into two subgroups based on whether they had an obesity diagnosis recorded in the Swedish National Patient Register before their inclusion in the study. We observed that individuals in the control subgroup with a prior obesity diagnosis had a lower life expectancy compared to those without an obesity diagnosis recorded before the study commenced. These findings, confirmed in the excess candidates from the SOS Matching Cohort, suggest that individuals with an obesity diagnosis captured in real-world datasets may be less healthy than those with obesity who volunteer for clinical studies. This disparity in health status could introduce significant bias in obesity studies using data from registers and electronic health records.

Less than 9% of the controls who volunteered to participate in the SOS study had a prior obesity diagnosis, reflecting the fact that obesity itself without comorbidities does not lead to hospitalization or specialist outpatient care. The higher risk profile of those with an obesity diagnosis in the control group of our study is in line with a Danish study where a majority of those diagnosed with obesity had co-morbidities such as heart disease, osteoarthritis and type 2 diabetes.[14] Thus, among individuals with an obesity diagnosis who do not undergo surgery, those with poor health are probably over-represented in real-world datasets. Conversely, the bariatric surgery groups created from real-world data may be healthier because they consist of individuals who opted to undergo surgical obesity treatment and have been deemed medically suitable for this procedure. Taken together, differences in health status between the surgery and control groups in retrospective cohorts may result in overestimation of the health benefits of bariatric surgery due to selection bias.

It is important to note that results from both the recent meta-analysis and the SOS study alone indicate that bariatric surgery increases life expectancy by several years, but it is unclear whether the meta-analysis provides the most correct estimate. With the exception of one study in which the control group consisted of individuals with a self-reported BMI of 35 or more when applying for a driving license,[15] the retrospective studies included in the meta-analysis used data generated in the healthcare system, such as electronic medical records and health registers.[5] This implies that most of the participants in the retrospective control groups have had an illness or problem that caused them to seek medical care. In the current analysis of SOS study data, median survival time was approximately three years shorter in the controls with a prior obesity diagnosis compared to those without such a diagnosis. Furthermore, the group that underwent bariatric surgery had longer survival compared to controls with a prior obesity diagnosis. This was also true when compared to controls without a prior obesity diagnosis, although the difference was significantly smaller. Since the control subgroup with a prior obesity diagnosis is analogous to control groups in retrospective studies using real-world data, our observations may partly explain the considerably smaller benefit of bariatric surgery in terms of life years gained in the matched prospective SOS study compared to the recent meta-analysis.[5, 7].

A key feature of the SOS study is its exceptionally long follow-up period. However, studies with extensive follow-up times often rely on treatment methods that are no longer widely used. Although newer anti-obesity medications, such as GLP-1 analogs, can lead to significant weight loss, they were introduced too recently to affect the present analysis,[16] which ends on December 31, 2020. Consequently, comparisons between subgroups of the control group are unaffected. Our study also includes older surgical techniques, some of which result in less weight loss and may underestimate the benefits of surgical obesity treatment. A strength of our study is the extensive information collected at baseline. In studies using real-world data, information about baseline characteristics is usually limited, and matching and adjustments are often restricted to a few variables such as age and sex, while information on BMI and other risk factors may be unavailable. Another notable aspect of this study is its use of comprehensive and high-quality health registers. The Swedish Cause of Death Register is an essentially complete register of all official (underlying) causes of death[12] and the Swedish National Patient Register has 99% coverage of inpatient care and around 80% coverage of specialist outpatient care visits.[10].

A limitation of our current comparison of controls with and without a prior obesity diagnosis is that all participants had to be eligible for surgery, which excluded potential controls with unacceptable surgical risks due to poor health. This implies that participants in the SOS control group with a prior obesity diagnosis may be healthier than the average person with an obesity diagnosis in the Swedish National Patient Register. It is therefore possible that we are underestimating the differences between controls with and without a prior obesity diagnosis, and the disparity in health status between surgery and control groups in studies compiled from digital health datasets could be even greater than indicated by our study.

The methods used for validating and introducing clinical treatments have followed a well-established and consistent pattern for several decades, with randomized controlled trials (RCTs) being the gold standard due to their minimized bias.[17] However, RCTs have disadvantages, including high costs and long recruitment times. As a result, alternatives like synthetic or external control arms[18, 19] are starting to emerge and are increasingly being considered by decision-makers.[20, 21] In externally controlled trials, outcomes after active treatment are compared with data from routine care involving a group of untreated individuals identified in health registers or electronic healthcare records. So far, external control arms have primarily been used to evaluate treatments for rare diseases and malignancies. However, using controls identified in real-world datasets to evaluate the effects of bariatric surgery can be seen as a modification of this study approach. The validity of the Swedish National Patient Register depends on whether a disease or procedure requires hospitalization. For serious diseases such as myocardial infarction, the register has high completeness, while it is much lower for conditions such as hypertension and type 2 diabetes that are mainly managed in primary care.[22] The results of our study may therefore have implications for externally controlled trials in fields beyond obesity, particularly for diseases where a significant proportion of affected individuals are not captured in registers. The source of real-world data used for identifying controls and the purpose of data collection can introduce various forms of selection bias. For example, health register data may identify controls who are generally less healthy, while driving license registers are likely to include healthier individuals. Consequently, it is essential to consider the characteristics of the included individuals carefully.

In conclusion, our results show that controls in the SOS study with a prior obesity diagnosis had higher mortality than those without such a diagnosis before recruitment to the study. Although bariatric surgery is an established treatment for severe obesity,[23] randomized studies demonstrating the long-term effects of this treatment on serious comorbidities and life expectancy are lacking. Several observational long-term studies have shown associations between bariatric surgery and reduced incidence of cardiovascular disease[24, 25 and cancer,^26^, ^27^, ^28^, ^29^] as well as reduced overall mortality.[5, 25, 29] Apart from the prospective matched SOS study, these results are based on studies involving individuals with obesity identified in real-world datasets. Controls in these studies are likely more analogous to the controls in the SOS study with, rather than without, a prior obesity diagnosis. Thus, the possibility that studies using real-world data may overestimate the benefits of bariatric surgery should be considered when individuals with obesity seek surgical treatment to lower the risk of obesity associated diseases and premature death.[30, 31] Similarily, selection bias could lead to an overestimation of the benefits of other obesity treatments in studies using controls identified by prior obesity diagnosis.

## Electronic supplementary material

Below is the link to the electronic supplementary material.

Supplementary Material 1
