# Supplementary material for: Possible selection bias in register-based obesity studies
Source: Eur J Epidemiol. 2025 May 12;40(7):759–66. doi: 10.1007/s10654-025-01237-6 (PMC12304022; doi:10.1007/s10654-025-01237-6)
Supplement: Supplementary file 1 — Supplementary Material 1 [file 10654_2025_1237_MOESM1_ESM.docx]

**Supplement to: Possible selection bias in register-based obesity studies**

Lena MS Carlsson (MD), Markku Peltonen (PhD), Peter Jacobson (MD), Johanna C. Andersson-Assarsson (PhD), Per-Arne Svensson (PhD), Magdalena Taube (PhD), Cecilia Karlsson (MD), Sofie Ahlin (MD), Felipe M Kristensson (MD), Rosie Perkins (PhD), Ida Arnetorp (student), Alexander Carlsson (student), Lucas Admeus (MD), Elin Langegård (BSc), Björn Carlsson (MD), Kajsa Sjöholm (PhD).

**Table of Contents**

Supplementary Figure S1. Flowchart Page 3

Supplementary Figure S2. Survival in the excess candidate pool Page 4

Supplementary Figure S3. Body mass index in the SOS surgery and control groups Page 5

Supplementary Figure S4. Survival in the SOS surgery and control subgroups Page 6

Supplementary Table S1. Baseline characteristics of the excess candidate pool Page 7

Supplementary Table S2. Mortality and life expectancy in the excess candidate pool Page 8

Supplementary Table S3. Causes of death Page 9

Supplementary Table S4. Baseline characteristics of the SOS surgery group Page 10

Supplementary Table S5**.** Mortality in the SOS control subgroups compared to the surgery group Page 11

**Supplementary Figure S1.** Flowchart illustrating the recruitment of the surgery and control groups of the SOS intervention study as well as the creation of subgroups, with and without prior obesity diagnosis in the Swedish National Patient Register, within the SOS control group . Individuals who participated in the matching examination but were not included in the intervention study constitute the excess candidate pool, which was used for verification of the results from the SOS control group in this study.

**Figure S2. Survival in the SOS excess candidate pool stratified by prior (before matching examination) obesity diagnosis in the Swedish National Patient Register.** Shown are Kaplan–Meier estimate of survival (opaque lines) and the estimate of survival from an unadjusted Gompertz regression model extrapolated up to 37 years (fainter lines).

**Supplementary Figure S3.** Body mass index over 10 years in the SOS surgery group and SOS control group stratified by prior (before study inclusion) obesity diagnosis in the Swedish National Patient Register.

**Supplementary Figure S4.** **Survival in the SOS surgery group compared to subgroups of the SOS control group with (A) and without (B) a prior obesity diagnosis in the Swedish National Patient Register before study inclusion.** Shown are Kaplan–Meier estimate of survival (opaque lines) and the estimate of survival from an unadjusted Gompertz regression model extrapolated up to 37 years (fainter lines).

**Supplementary Table S1.** Baseline characteristics of the excess candidate pool stratified by prior obesity diagnosis in the Swedish National Patient Register. Individuals with previous bariatric surgery are excluded.

|  | **Excess candidates with a prior obesity diagnosis**  (n=182) | **Excess candidates without a prior obesity diagnosis**  (n=2577) | p-value |
| --- | --- | --- | --- |
| Age - years | 48.7±5.9 | 48.0±6.0 | 0.138 |
| Male sex – no. (%) | 57 (31) | 1362 (53) | <0.001 |
| Body mass index – kg/m^2^ | 42.5±6.4 | 38.3±4.6 | <0.001 |
| Waist-to-hip ratio | 0.992±0.080 | 0.997±0.068 | 0.340 |
| Hypertension – no. (%)† | 136 (75) | 1816 (70) | 0.239 |
| Type 2 diabetes – no. (%)° | 40 (22) | 332 (13) | 0.001 |
| Blood glucose - mmol/L | 5.5±2.2 | 5.0±1.8 | 0.001 |
| Insulin – mU/L | 21.6±13.90 | 19.6±11.6 | <0.027 |
| HOMA-IR | 7.2±9.3 | 5.2±4.5 | <0.001 |
| HDL-cholesterol - mmol/L | 1.4±0.4 | 1.3±0.3 | 0.218 |
| Total-cholesterol – mmol/L | 5.7±1.4 | 5.9±1.1 | 0.064 |
| LDL-cholesterol – mmol/L | 3.3±1.1 | 3.5±1.0 | <0.001 |
| Triglycerides – mmol/L | 2.4±2.2 | 2.3±1.7 | 0.307 |
| Daily smoking – no. (%) | 58 (32) | 582 (23) | 0.006 |
| Alcohol consumption – g/day | 8.8±18.7 | 12.2±17.7 | 0.013 |
| Self-rated health status* | 4.3±1.3 | 3.9±1.4 | <0.001 |
| Cancer before baseline – no. (%) | 10 (5) | 99 (4) | 0.241 |
| Cardiovascular disease before baseline – no. (%) | 13 (7) | 101 (4) | 0.050 |

† Defined as diastolic blood pressure > 90 mm Hg, systolic blood pressure > 140 mm Hg, or self-reported antihypertensive medication.

°Based on fasting blood glucose level ≥ 6.1 mmol/L and/or use of anti-diabetes medication.

*Higher scores represent worse self-rated health status according to a graded scale (range 1–7).

**Supplementary Table S2.** Mortality rates, hazard ratios and differences in median life expectancy, from Gompertz proportional hazard regression model, in the excess candidate pool stratified by prior obesity diagnosis.

|  | **Excess candidates with a prior obesity diagnosis** | **Excess candidates without a prior obesity diagnosis** |
| --- | --- | --- |
| **n** | 182 | 2,577 |
| **Person-time (years)** | 3,260 | 55,196 |
| **Events** | 92 | 948 |
|  |  |  |
| **Mortality rate** | 28.2 | 17.2 |
| **95% CI** | 23.0-34.6 | 16.1-18.3 |
|  |  |  |
| **HR (unadj)** | 1.82 | Ref. |
| **95% CI** | 1.47-2.25 |  |
| **p-value** | <0.001 |  |
|  |  |  |
| **HR (adj#)** | 1.99 | Ref. |
| **95% CI** | 1.59-2.49 |  |
| **p-value** | <0.001 |  |
|  |  |  |
| **HR (full adj*)** | 1.57 | Ref. |
| **95% CI** | 1.25-1.98 |  |
| **p-value** | <0.001 |  |
|  |  |  |
| **Difference in median survival time, years (unadj)** | -6.1 | Ref. |
| **95% CI** | -8.3 to -3.9 |  |
|  |  |  |
| **Difference in median survival time, years (adj#)** | -6.7 | Ref. |
| **95% CI** | -8.9 to -4.6 |  |
|  |  |  |
| **Difference in median survival time, years (full adj*)** | -4.3 | Ref. |
| **95% CI** | -6.5 to -2.2 |  |

# Adjusted for age and sex.

* Adjusted for age, sex and BMI.

**Supplementary Table S3.** Causes of death in the SOS study control group stratified by prior (before study inclusion) obesity diagnosis in the Swedish National Patient Register.

|  | **Controls with a prior obesity diagnosis**  (n=177) | | **Controls without a prior obesity diagnosis**  (n=1863) | |
| --- | --- | --- | --- | --- |
| **CAUSES OF DEATH** | **Number of deaths** | **Mortality rate per 1000 persons years (95% CI)** | **Number of deaths** | **Mortality rate per 1000 persons years (95% CI)** |
| **CARDIOVASCULAR** | **27** | **8.0 (5.5-11.6)** | **223** | **5.6 (4.9-6.4)** |
| **Cardiac** | 21 |  | 188 |  |
| Myocardial infarction | 9 |  | 54 |  |
| Heart failure | 6 |  | 42 |  |
| Sudden death | 6 |  | 87 |  |
| Other cardiac | 0 |  | 5 |  |
| **Stroke** | 6 |  | 28 |  |
| Intracerebral hemorrhage | 3 |  | 10 |  |
| Infarction | 2 |  | 10 |  |
| Other or unspecified | 1 |  | 8 |  |
| **Large artery disease** | 0 |  | 7 |  |
|  |  |  |  |  |
| **MALIGNANCY** | **13** | **3.8 (2.2-6.6)** | **174** | **4.4 (3.8-5.1)** |
|  |  |  |  |  |
| **OTHER** | **27** | **8.0 (5.5-11.6)** | **177** | **4.4 (3.8-5.2)** |
| Complication after surgery | 1 |  | 5 |  |
| Infection | 7 |  | 61 |  |
| Neurological disease | 1 |  | 16 |  |
| Kidney disease | 2 |  | 11 |  |
| Liver disease | 0 |  | 5 |  |
| Gastrointestinal disease | 0 |  | 4 |  |
| Lung disease | 5 |  | 12 |  |
| Thromboembolic disease | 2 |  | 15 |  |
| Other or multiple conditions | 3 |  | 19 |  |
| Alcohol abuse | 0 |  | 9 |  |
| Trauma / Accident | 4 |  | 6 |  |
| Suicide | 1 |  | 2 |  |
| Unknown | 1 |  | 12 |  |
|  |  |  |  |  |
| **TOTAL** | **67** | **19.7 (15.5-25.1)** | **574** | **14.4 (13.3-15.7)** |

**Supplementary Table S4.** Baseline characteristics of the SOS surgery group.

|  | **Surgery group**  (n=2007) |
| --- | --- |
| Age – years | 47.2±5.9 |
| Male sex – no. (%) | 587 (29.2) |
| Body mass index – kg/m^2^ | 42.4±4.5 |
| Waist-to-hip ratio | 0.992±0.078 |
| Hypertension – no. (%)† | 1571 (78.4) |
| Type 2 diabetes – no. (%)° | 344 (17.2) |
| Blood glucose – mmol/L | 5.2±2.0 |
| Insulin – mU/L | 21.5±13.7 |
| HOMA-IR | 5.8±5.4 |
| HbA1c – mmol/mol | 42.4±12.7 |
| HDL-cholesterol – mmol/L | 1.4±0.3 |
| Total-cholesterol – mmol/L | 5.9±1.1 |
| LDL-cholesterol - mmol/L | 3.5±1.0 |
| Triglycerides – mmol/L | 2.2±1.5 |
| Daily smoking – no. (%) | 518 (25.8%) |
| Alcohol consumption – g/day | 5.2±7.2 |
| Self-rated health status* | 3.7±1.3 |
| Cancer before baseline – no. (%) | 25 (1.2) |
| Cardiovascular disease before baseline – no. (%) | 46 (2.3) |

† Defined as diastolic blood pressure > 90 mm Hg, systolic blood pressure > 140 mm Hg, or self-reported antihypertensive medication.

°Based on fasting blood glucose level ≥ 6.1 mmol/L and/or use of anti-diabetes medication.

*Higher scores represent worse self-rated health status according to a graded scale (range 1–7).

**Supplementary Table S5.** Mortality rates, hazard ratios and differences in median life expectancy, from Gompertz proportional hazard regression model, in the SOS study control group with and without a prior obesity diagnosis compared to the surgery group.

|  | **Controls with a prior obesity diagnosis** | **Controls without a prior obesity diagnosis** | **Surgery group** |
| --- | --- | --- | --- |
| **n** | 177 | 1863 | 2007 |
| **Person-time (years)** | 3,395 | 39,776 | 45,574 |
| **Events** | 67 | 574 | 559 |
|  |  |  |  |
| **Mortality rate** | 19.7 | 14.4 | 12.3 |
| **95% CI** | 15.5-25.1 | 13.3-15.7 | 11.3-13.3 |
|  |  |  |  |
| **HR (unadj,)** | 1.81 | 1.24 | Ref. |
| **95% CI** | 1.40-2.35 | 1.11-1.40 |  |
| **p-value** | <0.001 | <0.001 |  |
|  |  |  |  |
| **HR (adj#)** | 1.57 | 1.05 | Ref. |
| **95% CI** | 1.21-2.03 | 0.94-1.19 |  |
| **p-value** | 0.001 | 0.378 |  |
|  |  |  |  |
| **HR (adj*)** | 1.65 | 1.15 | Ref. |
| **95% CI** | 1.27-2.15 | 1.02-1.30 |  |
| **p-value** | 0.001 | 0.024 |  |
|  |  |  |  |
| **Difference in median survival time, years (unadj.)** | -5.3 | -2.0 | Ref. |
| **95% CI** | -7.7 to -3.0 | -3.0 to -0.9 |  |
|  |  |  |  |
| **Difference in median survival time, years (adj#)** | -3.8 | -0.5 | Ref. |
| **95% CI** | -6.0 to -1.6 | -1.5 to 0.5 |  |
|  |  |  |  |
| **Difference in median survival time, years (adj*)** | -4.3 | -1.2 | Ref. |
| **95% CI** | -6.5 to -2.1 | -2.3 to -0.2 |  |

# Adjusted for age and sex.

* Adjusted for age, sex and BMI.
